# Supplementary material for: Efficient screening of adsorbed receptors for Salmonella phage LP31 and identification of receptor-binding protein
Source: Microbiol Spectr. 2023 Sep 20;11(5):e02604-23. doi: 10.1128/spectrum.02604-23 (PMC10581130; doi:10.1128/spectrum.02604-23)
Supplement: Fig. S1 to S3 — Gel images of strain construction. [file spectrum.02604-23-s0001.docx]

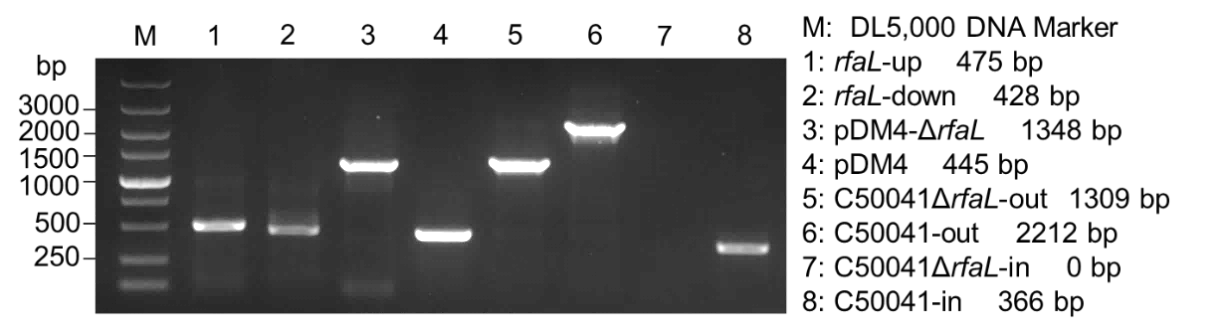


**FIG. S1** Identification of mutant C50041Δ*rfaL*^58-358^ by PCR. The lanes contain: PCR products for: (1) up- and (2) down-stream sequences of the *rfaL* gene using the primer pairs up-F/R and down-F/R, respectively. PCR products obtained from the recombinant plasmids (3) pDM4-*rfaL*^Δ58-358^ and (4) pDM4 the using primer pair pDM4-F/R. PCR products obtained from the genome of C50041Δ*rfaL*^58-358^ using (5) primer pair out-F/R and (7) primer pair in-F/R, respectively. PCR products obtained from the wild-type genome are also shown using (6) primer pair out-F/R and (8) primer pair in-F/R. (M) Molecular marker.


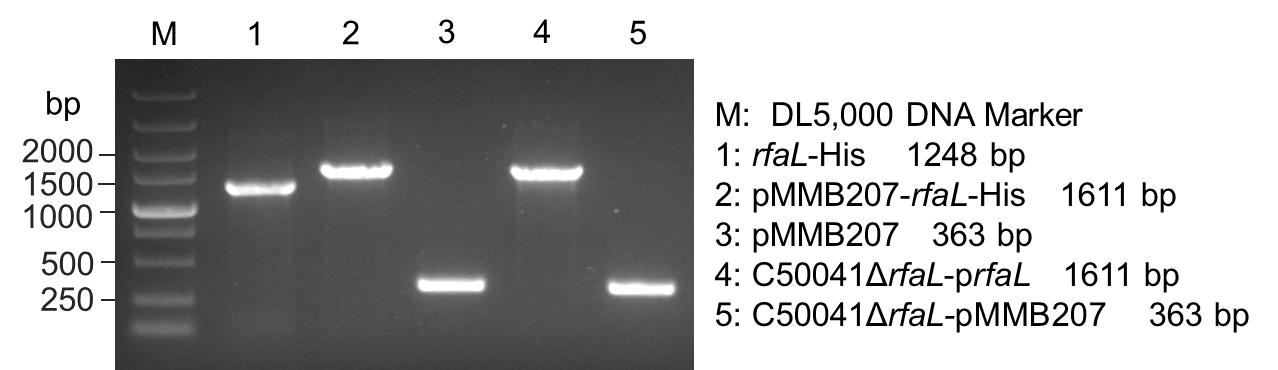


**FIG. S2** Identification of complemented strain C50041Δ*rfaL*^58-358^ carrying p*rfaL* (C50041Δ*rfaL*^58-358^-p*rfaL*) by PCR. Lanes contain PCR products for the *rfaL* gene (1) using primer pair *rfaL*-F/*-*his R. (2) PCR products for the recombinant, expressing plasmid pMMB207-*rfaL*-His and (3) pMMB207 using primer pair pMMB207-F/R. PCR products using primer pair pMMB207-F/R and (4) total C50041Δ*rfaL*^58-358^-p*rfaL* genome or (5) total DNA from C50041Δ*rfaL*^58-358^ carrying pMMB207 (C50041Δ*rfaL*^58-358^-pMMB207). (M) Molecular marker.


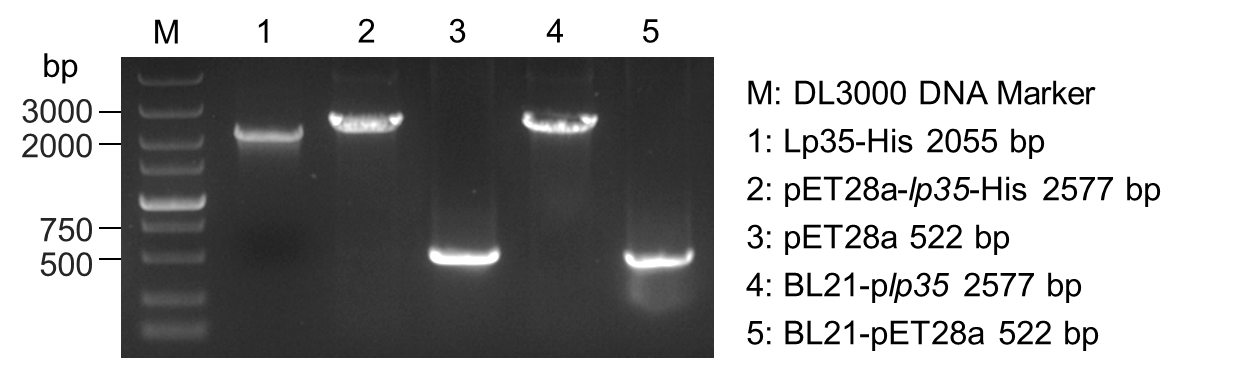


**FIG. S3** Identification of protein expression bacteria BL21 carrying p*lp35* (BL21-p*lp35*) by PCR. The lanes contain PCR products for the *lp35* gene using (1) primer pair *lp35*-F/R*-*his. PCR products for recombinant, expression plasmids (2) pET28a-*lp35*-His and (3) pET28a using primer pair pET28a-F/R. PCR products from the total DNA of (4) BL21-p*lp35* strain genome and (5) BL21 carrying pET28a (BL21- pET28a) using primer pair pET28a-F/R. (M) Molecular marker.
