# Supplementary material for: Efficient screening of adsorbed receptors for Salmonella phage LP31 and identification of receptor-binding protein
Source: Microbiol Spectr. 2023 Sep 20;11(5):e02604-23. doi: 10.1128/spectrum.02604-23 (PMC10581130; doi:10.1128/spectrum.02604-23)
Supplement: Table S1 — Primers designed for and used in this study. [file spectrum.02604-23-s0002.docx]

**TABLE S1.** Primers designed for and used in this study.

| **Primer name** | **Sequence (5’ → 3’)** |
| --- | --- |
| **Identification of transposon Insertion site** | |
| First round of PCR | |
| AB1 | GGCCACGCGTCGACTAGTACNNNNNNNNNNACGCC |
| AB2 | GGCCACGCGTCGACTAGTACNNNNNNNNNNCCTGG |
| AB3 | GGCCACGCGTCGACTAGTACNNNNNNNNNNCCTCG |
| SP1 | GCTGACCGCTTCCTCGTGCTTTACG |
| Second round of PCR | |
| ABS | GGCCACGCGTCGACTAGTAC |
| SP2 | CATCGCCTTCTATCGCCTTCTTGAC |
| Sequencing | |
| pSC189-seq | CGCGAAGTTCCTATTCCGAAGTTCC |
| ***rfaL* Gene deletion** | |
| Up-F | GAGCGGATAACAATTTGTGGAATCCCGGGACGAAGGCTTTGACTATGTGGAT |
| Up-R | GGCTTATCTCCGGTGAGCGTGAGACCTGATAAATC |
| Down-F | ACGCTCACCGGAGATAAGCCCCTACAATGCTCATC |
| Down-R | AGCGGAGTGTATATCAAGCTTATCGATACCCCAGCAAAAAAGGGGGGATTAG |
| In-F | GAAACAAAAGAAACGGTTGCGAA |
| In-R | GGCTAATAAAATGGCACCAACTC |
| Out-F | CCTGGGATACGATAAACCGCAGT |
| Out-R | GACGCCGCAAAAGAGATTGGAAC |
| pDM4-F | GGTGCTCCAGTGGCTTCTGTTTCTA |
| pDM4-R | CAGCAACTTAAATAGCCTCTAAGGT |
| ***rfaL* Gene complement** | |
| *rfaL*-F | CTCGGTACCCGGGGATCCTCTAGACTAAAGGAAGACGTTATGCTAACCACATCATTAACGT |
| *rfaL*-His-R | TCATCCGCCAAAACAGCCAAGCTTTAGTGATGATGATGATGATGTCTATTTCTTAGCGCCAACAG |
| pMMB207-F | CTCCCGTTCTGGATAATGTT |
| pMMB207-R | GGCGTTTCACTTCTGAGTTCG |
| **Protein expression** | |
| *lp35*-F | TAAGAAGGAGATATACCATGTCTAGTGGTTGCGGTGAGG |
| *lp35*-His-R | GTGGTGGTGGTGGTGCTCGAGTTATGCCAAAGTTAATCTTGTGTAGCT |
| *Lp24*-F | TAAGAAGGAGATATACCATGGCGTTACAACCATATAAGGG |
| *Lp24*-His-R | GTGGTGGTGGTGGTGCTCGAGGGCGTACTTAATGCGCTGGA |
| *lp34*-F | TAAGAAGGAGATATACCATGGCGTTACAACCATATAAGGG |
| *lp34*-His-R | GTGGTGGTGGTGGTGCTCGAGGGCGTACTTAATGCGCTGGA |
| pET28a-F | CACCATACCCACGCCGAAAC |
| pET28a-R | AAAAACCCCTCAAGACCCGT |
